# Quantitative measurements of aerosols from air-polishing and ultrasonic devices: (How) can we protect ourselves?

**DOI:** 10.1371/journal.pone.0244020

**Published:** 2020-12-15

**Authors:** Manuela Kaufmann, Alex Solderer, Andrea Gubler, Florian J. Wegehaupt, Thomas Attin, Patrick R. Schmidlin

**Affiliations:** Clinic of Conservative and Preventive Dentistry, Center of Dental Medicine, University of Zurich, Zurich, Switzerland; Klinikum der Johann Wolfgang Goethe-Universitat Frankfurt Klinik fur Nuklearmedizin, GERMANY

## Abstract

**Aim:**

To assess the distribution and deposition of aerosols during simulated periodontal therapy.

**Methods:**

A manikin with simulated fluorescein salivation was treated by four experienced dentists applying two different periodontal treatment options, i.e. air-polishing with an airflow device or ultrasonic scaling in the upper and lower anterior front for 5 minutes, respectively. Aerosol deposition was quantitatively measured on 21 pre-defined locations with varying distances to the manikins mouth in triplicates using absorbent filter papers.

**Results:**

The selected periodontal interventions resulted in different contamination levels around the patient’s mouth. The highest contamination could be measured on probes on the patient’s chest and forehead but also on the practitioner’s glove. With increasing distance to the working site contamination of the probes decreased with both devices. Air-polishing led to greater contamination than ultrasonic.

**Conclusion:**

Both devices showed contamination of the nearby structures, less contamination was detected when using the ultrasonic. Affirming the value of wearing protective equipment we support the need for universal barrier precautions and effective routine infection control in dental practice.

## Introduction

Due to the highly infective pandemic threat of COVID-19, several questions regarding prophylactic and therapeutic applications during medical and dental interventions, especially due to aerosols, have arisen. Notably dentists, dental hygienists and dental assistants represent a professional group, who is among at the greatest risk for exposure to bacteria and viruses, since the oral cavity holds a high potential of transmissibility and susceptibility to this and other infectious agents, especially, when aerosol producing instruments are used [1].

Pathogens can be transmitted through inhalation of airborne microorganisms, which can remain suspended in the air for several hours as well as contact of conjunctival, nasal, or oral mucosa with droplets and aerosols containing microorganisms generated from an infected individual and propelled a short distance by coughing and talking without protection or aerosol producing dental treatments [2]. Many dental procedures lead to aerosols, droplets and rebound spray—a mix of cooling spray and saliva—that are contaminated with bacteria, viruses, debris and/or blood. With regard to COVID-19, the salivary viral load was found highest during the first week after symptom onset but could be detected until 25 days after [3]. One study investigating SARS-CoV-2 found that viral load was consistently high in the saliva and relatively higher than in the oropharynx during the early stage [4]. In a hospital setting with health care workers exposed to high risk procedures, a risk ratio (RR) of 2.5 was detected for acquiring viral or bacterial infection [5]. Dental care varies in every country due to different regulatory guidelines and regulations. Respective hygiene concepts may therefore vary, however, efforts are aimed at minimizing airborne contaminations whenever possible [6].

Up today, the protection of the dental personnel and the patient, especially immunocompromised patients, during the pandemic outbreak of COVID-19, has led to a distinct restriction of dental practice treatments in Switzerland during the lockdown phase. Even the first weeks afterwards, ultrasonic treatments and therapy using air-abrasive devices, were strictly forbidden, but not based on scientific ground.

Several studies assessed already aerosol distribution, the efficacy of evacuation systems and the adjunct application of antimicrobial and -viral substances to reduce the pathogenic load [7]. The majority of the studies assessed different devices and personal protective equipment and found in the majority of the studies a contamination with bacterial and/or viral aerosols. However, according to the authors’ knowledge, quantitative studies on aerosol protection assessing the distribution and efficacy of specific protection devices including FFP2 (KN95) masks are still scarce. Therefore, this study analysed the spreading of oral aerosols, droplets and rebound spray focusing on two different frequently used periodontal procedures, i.e. air-polishing and ultrasonic debridement, which are known (or supposed) causing aerosols applying for the first time a saliva-contamination surrogate model. In this way, we were able to detect the contamination around the patient and protective potential of a given sample of protective measures in this context. The authors hypothesized that the areas around or close to the mouth were more prone to accumulate measurable amounts of contaminated or dyed saliva derived from the delivered spraying and that the protection aids effectively protect the team.

## Material and methods

### Experimental set-up

**Fig 1:**
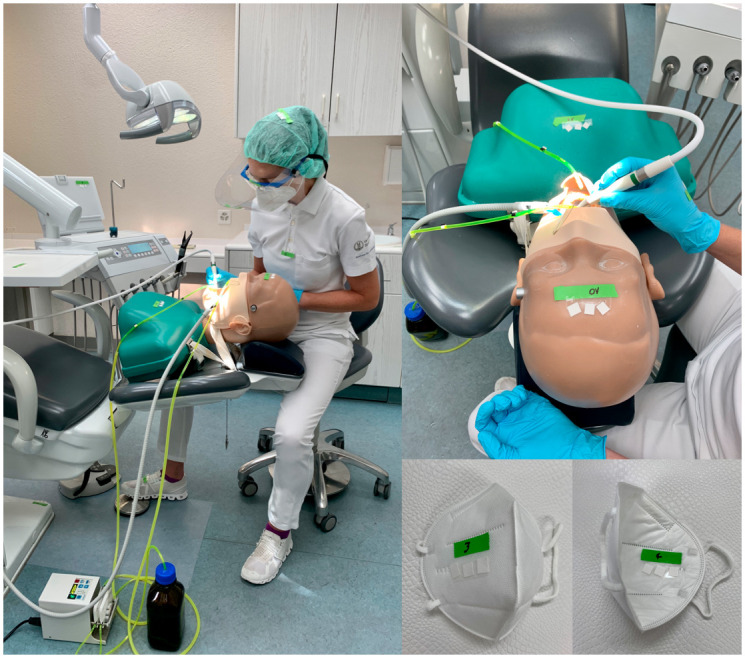
Depiction of the experimental set-up, the dental unit and the exemplified demonstration of adsorbent filters and their fixation via double-sided adhesive tapes.

A manikin head was mounted in a dental unit chair (Kavo Estetica E70, Kavo Dental AG, Kloten, Schweiz) in a standard reclining position mimicking a person of approximately 175 cm height. The overall set-up is illustrated in Fig 1: In order to mimic realistic simulation of contaminated saliva, a fluorescent fluorescein solution (100ppm fluorescein (100 mg/L) in deionized H_2_0) was dripped into the working area with a constant flow rate of 4 ml per minute using a tube pump (Ismatec, Modell ISM834C, Cole-Parmer GmbH, Wertheim, Germany) ensuring a constant flow rate.

White square filter paper sheets (Whatman^®^Benchkote^®^ surface protector, WHA2300916, Sigma Aldrich resp Merck KGaA, Darmstadt, Germany; 1x1 cm) were fixed with adhesive tapes at pre-defined locations of interest (Table 1).

**Table 1:** Overview of the different locations, where the aerosols were collected and the respective distances of the treatment area.

Number	Location	Distance to patients’ mouth	Airflow			Ultrasonic		
**1**	Positive control (soaked with fluorescent)	0			12			12
**2**	Patient chest	15–25 cm	2		10	8	3	1
**3**	Practitioner glove right or left backhand-middle	15–25 cm	7	1	4	11	1	
**4**	Practitioner protective shield outside	30 cm	10	1	1	11	1	
**5**	Patient forehead	20 cm	6	1	5	9	3	
**6**	Patient chair base	120 cm	9	2	1	10	2	
**7**	Patient chair tray	30 cm	8	3	1	11	1	
**8**	Practitioner shirt chest	15–30 cm	11	1		9	2	1
**9**	Patient chair spittoon	70 cm	9	2	1	9	3	
**10**	Practitioner protective goggles outside	30 cm	10	1	1	10	2	
**11**	Practitioner shoe at the top in the middle	110 cm	10	2		10	1	1
**12**	Patient chair x-ray board	50 cm	10	2		10	2	
**13**	Instrument boy	2 m	8	4		11	1	
**14**	Patient chair lamp	50 cm	11	1		11	1	
**15**	Practitioner KN95 outside	25–30 cm	10	2		11	1	
**16**	Practitioner KN95 inside	25–30 cm	11	1		11	1	
**17**	Practitioner protective shield inside	30 cm	12			11	1	
**18**	Practitioner protective goggles inside	30 cm	11	1		11	1	
**19**	Cupboard surface	2 m	9	3		12		
**20**	Practitioner surgical cap at the top in the middle	50–70 cm	12			12		
**21**	Negative control (soaked with pure unit water)	0	12			12		

Results shown in a traffic-light system according to the nL-amount of the detected fluorescein (red: > 20 nL, orange: 10–20 nL, green: < 10 nL).

Three paper sheets were set in each place (Tesa^®^ double-sided adhesive tape 12mm x 7,5m) for each run and a code was assigned depending on the position. Fluorescein (resorcinol phthalein (C_20_H_12_0_5_), melting point -320°C, molecular weight -332.31), an orange-red odourless powder was used as surrogate marker dissolved in liquid. It was procured from Sigma Aldrich (resp. Merck KGaA, Darmstadt, Germany) diluted and prepared in a bottle from which tubes pulled straight out of the bottle to the pump regulating the flow rate. Cotton rolls were used in the lower jaw to provide proper working space.

During the experiment, any excess water, powder or splatter was sucked off by a cheek saliva ejector with a diameter of 0.5 cm, but no HVE (high-volume evacuator) was used—simulating patient treatment without any assistance in a “worst-case” scenario with assumed higher aerosol formation.

After the treatment probes were put into 2ml Eppendorf tubes and 0.5 ml of Milli-Q water was added. After an incubation period of 1 hour on the shaker, photometric measurements were undertaken. Samples were measured in 96 well plates for fluorescence-based assays (M33089, Thermo Fisher Scientific, Waltham, MA USA) in a Plate Reader (Spectra Max M2, Bucher Biotec AG, Basel, Switzerland). 490nm was used as excitation wavelength and 525nm as emission wavelength. Samples were then quantified by using a standard curve (1ppm-0.001ppm Fluorescein).

### Simulated periodontal therapy and protection devices

For this study, two commonly used powered cleaning devices were used, i.e. an air-polishing device (AirFlow^®^-One, EMS, Nyon, Switzerland) and a piezoelectric ultrasonic scaler on a Kavo Estetica E70 treatment unit (PIEZO Scaler Tip 201, Kavo, REF 1.007.4024, Kavo Dental AG, Kloten, Switzerland).

For the air-polishing, distilled water and an erythritol-powder with a particle size of 14 μm, pH 7 and neutral taste was used (EMS, AirFlow^®^ PLUS powder). The compressed air was made available via the treatment unit with a pressure of 4 bar at a mains supply voltage of 230 VAC. Mock air polishing was performed at maximum water flow rate and step 9/10 powder regulation. Mock scaling was performed with the ultrasonic scaler set at medium power. Both test devices were applied in triplicates by four experienced dentists (one female and three males, three right-handed, one left-handed).

As protection materials, a KN95 mask (Haining jinbali Technology Co., Ltd. China GB2626-2006), safety glasses with side protection, and a protection shield were used as well as a surgical cap.

Treatments were performed for 5 minutes on the lower anterior teeth followed by 5 minutes on the upper anterior teeth of the manikin head. The authors simulated a possible worst-case scenario of splatter and aerosol dissemination assuming that working in the anterior teeth-area will lead to a higher outflow rate. After each 10 minute-run, the filter paper samples were immediately collected, the whole setting area cleaned up and new filter paper sheets were placed. The closed room was ventilated for 15 minutes. New filter papers were kept in a sealed box, not reachable for any contamination and were handled with clean tweezers only.

Three samples per location (21) were retrieved. To increase flourescein sensitivity, the three samples for every pre-defined location were measured together. nL-amount of the fluorescein was given in thirds: in total twelve end-sample results (4 dentists x 3 gateways) per location.

## Results

In total, 1512 samples were retrieved and analysed. Table 1 shows the descriptive statistics. A traffic light system was used to highlight the differences between the various located samples. Green indicates a measured amount of less than 10 nL, orange 10 to 20 nL and red more than 20 nL fluorescein distributed in total on all three filter paper sheets together per gateway.

**Fig 2:**
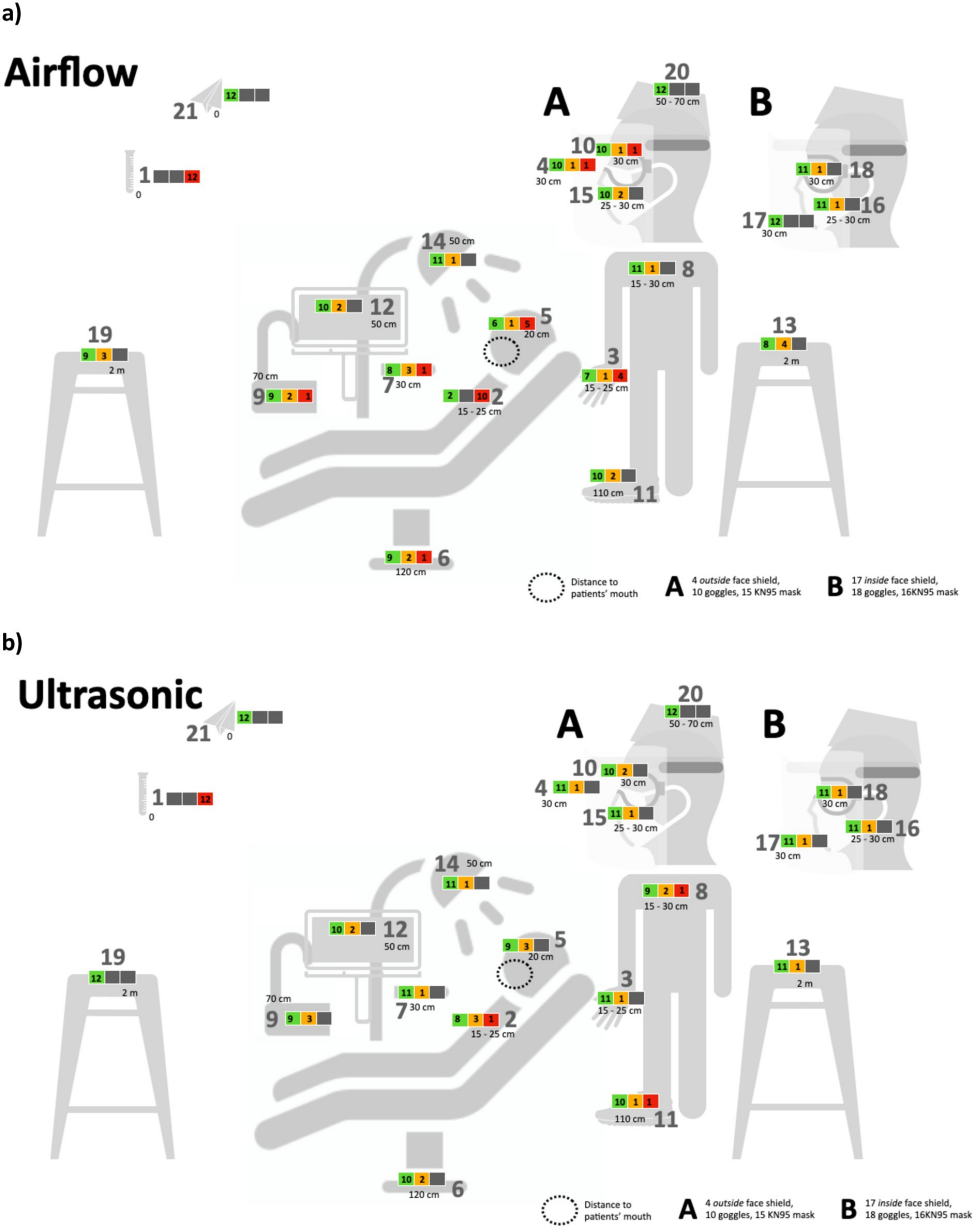
Clinical practice set-up and sample localization. **Contamination in respect to the location is shown in a small traffic-light (according to** Table 1**)**. a) Airflow b) Ultrasonic.

The amount of contamination of the set 21 points (Fig 2) varied with distance and device. The findings after the evaluation were:

1. Irrespective of the practitioner’s handedness, most fluorescein could be measured on probe in the middle of the patient’s chest and forehead but also on the practitioner’s glove backhand-middle when using the air polishing device.
2. With increasing distance to the working site (patient’s mouth) contamination of the probes decreased with both devices.
3. Air-polishing causes more contamination than ultrasonic, especially regarding the patients’ chest, practitioners’ gloves and patients’ forehead.
4. Some contamination was found outside but also inside the KN95 mask with all practitioners with both devices.
5. Practitioners’ clothes (shoe, shirt, cap, gloves) always get contaminated.

## Discussion

Daily practice will have to change as a result of the Covid-19 pandemic at least in the short term as aerosol-generating procedures (AGPs) are prevalent in dentistry. Guidelines for public health demand for evidence-based study results. Guidelines for public health demand for evidence-based study results. Evidence suggests that viral emerging diseases such as SARS-CoV-2, SARS, MERS, HIV, HCV, influenza or bacterial diseases such as tuberculosis have airborne transmission potential [8–12], but especially in the case of COVID-19 evidence of airborne transmission is mixed and it remains unclear whether people with COVID-19 produce enough virus-loaded aerosols to constitute a risk without talking, coughing or sneezing [13]. Most dental procedures using mechanical instrumentation such as air-abrasive devices, ultrasonic scalers or dental handpieces produce airborne particles i.e., those with aerodynamic diameters of 50 μm or less [14]. Material from the operative site becomes aerosolized by the action whereas noticed by the dental personnel or patient is only the water spray portion of the aerosol. Larger particles drop quicker to the floor or other nearby surfaces. Smaller particles can remain airborne longer and therefore be inhaled by dental personnel, patients or persons nearby.

The aim of this study was to investigate and assess the degree of spreading of aerosols around a dental practice unit when using two different mechanical periodontal instruments and working in an exposed zone as the anterior front teeth. Furthermore, it was shown to what extent the protective materials used in a typical dental practice set-up actually protect independently on whether a virus is loaded in the aerosol.

In the present study a manikin was used in order to create a standardized set-up with a predefined amount of flourescein, simulating a stimulated physiological saliva flow rate. Again, by the choice of the flow rate a worst-case scenario was drawn. Excess of water, powder or splatter was sucked off by a simple cheek saliva ejector. Hereby patient treatment without any assistance, as encountered in ordinary dental hygiene treatments, was simulated on purpose to simulate a “worst-case” scenario with assumed higher aerosol formation. The additional use of high-volume evacuation would have potentially minimized aerosol generation and contamination of the samples [15].

The current study was designed as a first study authors have decided to choose five minutes per jaw. The time span of ten minutes has been judged as appropriate in terms of practicability and significance. Further, through longer time spans soaking capacity of filters could have been a potential bias for the results.

The main advantage of the herein described method is the relatively simple handling. In addition, the filter papers show a high absorptive capacity and allow for a simple extraction of the Fluorescein afterwards. A disadvantage is the potential of accidental contamination. In this context, tweezers used to handle the samples should be carefully cleaned after each sample to avoid any (cross)contamination. This leads to additional time-consuming steps e.g. placing the samples on manikin and other surfaces.

It was shown that air-polishing led to a clearly higher amount of splattering in the near surroundings and showed also a slight tendency to a higher contamination of more distant structures. Our findings of splatter-distribution agree with other studies [15, 16], finding heavy contamination on the patient’s chest, although these studies focused on microbial (not viral) contamination. Overall the applied protective equipment ensured to shield the practitioner from heavy splattering. But as proven in the present study, also good equipment and a professional set-up may not protect from human neglect. The finest aerosols were not apparent in light streaming through the room, but fluorescein could still be detected in a distance > 2 m from the working site. Therefore, additionally to the equipment, the functional teamwork (an instructed dental assistant guiding the HVE correctly) and an enlightened dentist who not only treats responsibly, but also deals responsibly and correctly with protective material—for self-protection, but also protection against others—is required. Regarding causes leading to detectable fluorescent inside the mask in two samples of the present study, possible explanations could be aerosol clouds remaining in the operatory room for up to 30 minutes, as shown in previous studies [17, 18]. Therefore, it is recommended that the mask should not be removed immediately after the procedure in order to reduce the risk of contact with aerosols [17]. A second cause could be the filter capacity of KN95 masks filter amounting up to 95%, if worn correctly. It was further shown that the efficacy of the mask depends on the fit, the proper positioning, possible movements by the wearer and even the presence of facial hair [17]. Last, human neglect during the trials can never be totally excluded. Therefore, additional research is still needed to evaluate the role of filter capacity, proper use and handling.

Appropriate universal dental practice precautions findings whenever an aerosol is produced are:

- antiseptic rinsing before treatment [19–22]
- use of dental rubber dam [16, 23, 24]
- high-volume evacuation (HVE) suction device [14, 15, 25–27] or other dry-field isolation systems such as the “Isolite-system” [28]
- Barrier protection: mask, glove, eye protection goggle, protection shield [29]
- High-efficiency particulate air room filters

Further, dentists must always be aware that certain infected persons, called “super-spreaders”, produce many more aerosol particles than other persons, even without any treatment done [30]. Additionally, even in the complete absence of cooling water there is aerosolization of material from the operative site [25]. Regarding self-protection—special attention should be paid to the correct working posture, correct wearing of the protective mask, googles and shield. Manufacturers suggest that fluid resistant surgical facemasks filter 62% of airborne particles compared to 94% for FFP2 masks and 99% for FFP3 masks. In this experiment a KN95 mask was used (China GB2626-2006), which is equivalent to a FFP2 mask (EN 149–2001) but even filters 95% (3M Technical Bulletin May 2020). Should a mask be worn not close-fitted or goggles with side- and top-shields are placed at a distance from the face in order the glasses do not tarnish—aerosols will find a way to enter easily. Further, a wrong working posture will lead to aerosols getting behind a protective shield as used in our experiment. Protective shields with a closed-top design might protect more effectively. As the practitioners’ gloves become contaminated ubiquitously during the treatments, special attention should also be paid to what is handled and where during the treatment if the gloves are not disinfected or changed occasionally. Authors have decided to not use a gown in the experimental set-up, as it is not intended as regular protection device during oral hygiene procedures at the UZH (University of Zurich).

Different limitations of the current study can be pointed out. First of all, viral load was simulated through flourescein, due to easier and unriskfull handling for the authors. Authors can not predict infectious potential of measured flourescein amounts, when replaced through real virus. Due to ethical matters, however, manikins were used instead of real human patients, marking a second limitation. Furthermore, only devices for periodontal and oral hygiene treatments have been used in the current study. Additional research should evaluate the possible contamination caused by other dental devices.

Within the limitations of the current study, based on the results the authors judge both treatment modalities as safe if appropriate protective equipment is worn. In general, there is a strong need for adequate infection protocol principles during all treatments as infectious diseases could endanger all dental personnel and patients.

## Conclusion

Both devices showed contamination of the nearby structures with clearly less contamination when using the ultrasonic. Affirming the value of wearing protective equipment we support the need for barrier precautions and effective routine infection control in dental practice.
